# Testing multiple dose combinations in clinical trials

**DOI:** 10.1177/0962280219871969

**Published:** 2019-09-24

**Authors:** Saswati Saha, Werner Brannath, Björn Bornkamp

**Affiliations:** 1 Competence Centre for Clinical Trials, University of Bremen, Germany; 2 Novartis Pharma, Basel, Switzerland

**Keywords:** Drug combination, multiple testing, factorial design, intersection union tests

## Abstract

Drug combination trials are often motivated by the fact that individual drugs target the same disease but via different routes. A combination of such drugs may then have an overall better effect than the individual treatments which has to be verified by clinical trials. Several statistical methods have been explored that discuss the problem of comparing a fixed-dose combination therapy to each of its components. But an extension of these approaches to multiple dose combinations can be difficult and is not yet fully investigated. In this paper, we propose two approaches by which one can provide confirmatory assurance with familywise error rate control, that the combination of two drugs at differing doses is more effective than either component doses alone. These approaches involve multiple comparisons in multilevel factorial designs where the type 1 error can be controlled first, by bootstrapping tests, and second, by considering the least favorable null configurations for a family of union intersection tests. The main advantage of the new approaches is that their implementation is simple. The implementation of these new approaches is illustrated with a real data example from a blood pressure reduction trial. Extensive simulations are also conducted to evaluate the new approaches and benchmark them with existing ones. We also present an illustration of the relationship between the different approaches. We observed that the bootstrap provided some power advantages over the other approaches with the disadvantage that there may be some error rate inflation for small sample sizes.

## 1 Introduction

Combining different drugs is an important treatment option in many therapeutic areas such as respiratory, cardiovascular disease, cancer or infectious diseases. The hope is that by combining two drugs (with typically different modes of action), one can achieve a greater beneficial effect than either therapy alone. According to the regulatory requirement by the U.S. Food and Drug Administration's policy (21 CFR 300.50,^1^ CDER, 2013^2^), the fixed-dose combination drug must have confirmatory evidence for being more effective than each component drug alone. The primary questions that often arise in a drug combination therapy trial are: (1) Does there exist a dose combination that shows a better effect than the placebo control (effectiveness)? (2) Does there exist a dose combination that is superior to the individual treatments (superiority), where the individual treatments are often termed as monotherapies? (3) What are the specific combinations that fulfill both effectiveness and superiority?

Laska and Meisner^3^ and Snapinn^4^ are the first to consider the problem of testing the superiority of a certain combination treatment over the component treatments in a single dose combination setting. They conducted a “min-test”, where the minimum of the test statistics comparing the combination treatment with the monotherapies are used to show that the combination treatment is better. Extending the above approach in a multiple dose combination setting is not simple. In a multiple dose combination trial, multi-level factorial designs involving simultaneous multiple dose combination comparison comes into play and one has to propose multiple testing procedure (MTP) to address the multiplicity issues here.

Several authors have addressed the primary questions stated in the earlier paragraph in several ways. Hung^5–7^ proposed two single-step testing procedures that showed that there exists at least one combination in a multiple dose factorial drug combination study that is better than administering the component drugs alone. Hellmich and Lehmacher^8^ proposed a testing method for identifying the set of all minimum effective combinations in the case of monotone mean responses. Buchheister and Lehmacher^9^ proposed a closed testing procedure using special linear contrast tests and extended the global maximum test by Hung^6^ to a local maximum test for the identification of the superior dose combinations while preserving the family-wise error rate. Soulakova and Sampson^10^ and Soulakova^11^ proposed a procedure where their objective was to identify the set of minimum effective combination doses using the global average tests proposed by Hung^5^ under a closed testing principle. In all of these papers, the problems of efficacy and superiority of a combination drug were addressed separately which allowed explicit discussion of statistical issues. However, in practice, if a certain effective combination is shown to be superior, it is necessary to explain how results of the two individual procedures may be combined and what adjustments are needed to claim both efficacy and superiority. To address this, Soulakova^12,13^ expressed the problem of identifying the effective and superior drug combinations as a two stage problem, where the min-test is conducted at the first stage for comparing the drug combinations with the monotherapies at each dose level and then Holm's rejective multiple testing approach^14^ is employed in the second stage to obtain doses that show superiority over placebo.

In this paper, we focus on showing superiority with regard to the monotherapies and ignore the formal requirement of showing effectiveness, i.e. superiority over placebo. However, we will indicate in the discussion how to include the tests for effectiveness in some of the approaches considered here. We have already seen that many approaches are suggested in the literature to address the goals of testing superiority of drug combinations in a multi-level factorial design and identifying the set of superior dose combinations. The method suggested by Hung^7^ is one of the pioneer approaches that proposed a global test to deal with the above problems. Other authors have mainly proposed alternatives to test the same global null hypothesis that there exists at least one combination that provides superiority. Some authors proposed alternative approaches^9,15^ to control the family-wise error rate (FWER) strongly and identify all beneficial combinations in a multiple dose combination setting. However, most of the approaches proposed are either based on step-wise MTPs for a nonhierarchical hypothesis family, such as methods by Holm,^14^ Hochberg,^16^ and resampling methods by Westfall and Young^17^; see Soulakova^12,13^ or rely on closed testing principles proposed by Marcus, Peritz, and Gabriel^18^; also see Hellmich and Lehmacher,^8^ Buchheister and Lehmacher,^9^ Soulakova and Sampson,^10^ and Soulakova.^11^ In this article, instead of relying on conventional MTPs mentioned earlier (like Holm^14^ or Hochberg^16^), we will propose two new multiple testing procedures, by which one can test for superiority of the drug combination using: (*i*) a parametric bootstrap approach and (*ii*) FWER control under the least favorable null configuration. The parametric bootstrap approach suggested here estimates the parameters from the given dataset under the constraints imposed by the null hypothesis and obtains the null distribution of the test statistics by sampling data with the estimated parameters. Hence, it provides a tool to carry out the multiple comparisons in a parametric setup, without worrying about all the sampling distributions of the inherent test statistics in the composite null hypotheses. The least favorable null configuration approach aims to control the maximum type 1 error rate in the above multiple testing problem. It identifies the worst possible configurations (a subset of the null parameter space) that allow one to obtain a bound on the size of the test and control it within the desired significance limit. Thus the bootstrap approach and the least favorable null configuration approach both suggest ways of controlling the FWER which is a mandatory requirement for therapeutic dose response studies in Phase III clinical trials. While the least favorable null configuration approach leads to a FWER that is always below the nominal level *α* (and often much smaller), the bootstrap approach controls the FWER only asymptotically. However, the latter has the advantage of being less conservative.

## 2 Problem

Consider a random vector Y, containing the clinical measurement of interest and a (*r* + 1) × (*s* + 1) factorial design trial where the dose levels are coded as 0, 1, 2,…*r* for drug *A* and 0, 1, 2,…*s* for drug *B*. The response Y is observed for (*r* + 1) × (*s* + 1) parallel dose combination groups and it is assumed to have the following model

(1) $$Y_{\mathrm{ijk}}=\mu_{\mathrm{ij}}+{ɛ}_{\mathrm{ijk}}$$

where *k* = 1, 2,…*n*_*ij*_, *i* = 0, 1,…*r* and *j* = 0, 1,…*s*.

Here {*μ*_*i*0_, *i* = 1, 2,…*r*} are the mean responses of the monotherapies of Drug A and {*μ*_0*j*_, *j* = 1, 2,…*s*} are the mean responses of the monotherapies of Drug B and {*μ*_*ij*_, *i* = 1, 2,…*r, j* = 1, 2,…*s*} denote the mean response at dose combination, dose *i* of drug *A* and dose *j* of drug *B*. We assume here that ${ɛ}_{\mathrm{ijk}}∼\text{i}.\text{i}.\text{d}.N(0,\sigma^{2})$.

For the (*i, j*)th dose combination, the alternative hypothesis of interest is that the dose combination is more effective than both monotherapies, i.e. *H*_1*ij*_: *μ*_*ij*_ > *μ*_*i*0_ and *μ*_*ij*_ > *μ*_0*j*_. The corresponding null hypothesis is *H*_0*ij*_: *μ*_*ij*_ ≤ *μ*_*i*0_ or *μ*_*ij*_ ≤ *μ*_0*j*_. The global hypothesis associated with testing all active dose combinations versus their respective components is

(2) $$\begin{array}{c} H_{0}:\forall i,j;\mu_{\mathrm{ij}}\leq \mu_{i0}\;\text{or}\;\mu_{\mathrm{ij}}\leq \mu_{0j}\\ H_{1}:\exists i,j;\mu_{\mathrm{ij}}>\mu_{i0}\;\text{ and }\;\mu_{\mathrm{ij}}>\mu_{0j} \end{array}$$

## 3 Methods

### 3.1 Max-min test

We can rewrite the global null and corresponding alternative of the hypotheses discussed in equation (2) as

(3) $$\begin{array}{c} H_{0}:\cap_{i,j}H_{0\mathrm{ij}}\;\text{ where }H_{0\mathrm{ij}}:\mu_{\mathrm{ij}}-\mu_{i0}\leq 0\vee \mu_{\mathrm{ij}}-\mu_{0j}\leq 0\\ H_{1}:\cup_{i,j}H_{1\mathrm{ij}}\;\text{ where }H_{1\mathrm{ij}}:\mu_{\mathrm{ij}}-\mu_{i0}>0\wedge \mu_{\mathrm{ij}}-\mu_{0j}>0 \end{array}$$

If *T*_*ij*_ denotes the test statistic for testing *H*_0*ij*_ against *H*_1*ij*_, then the test statistic for testing the global null is given by

(4) $$T=\max_{i,j}T_{\mathrm{ij}}=\max_{i,j}\{\min\{T_{\mathrm{ij}}^{A},T_{\mathrm{ij}}^{B}\}\}$$

where $T_{\mathrm{ij}}^{A}$ and $T_{\mathrm{ij}}^{B}$ are the contrast test statistics used for testing whether drug combination is superior to the monotherapies with respect to Drug A and Drug B, respectively. As suggested by Hung^7^ and Soulakova,^12^ a simple approach here is to compute the *p*-value for each *T*_*ij*_ using the minimum of the test statistics $T_{\mathrm{ij}}^{A}$ and $T_{\mathrm{ij}}^{B}$. The distribution of $T_{\mathrm{ij}}^{k},k=A,B$ is given below

$$T_{\mathrm{ij}}^{A}=\frac{c'ij_{A}\bar{Y}}{\sqrt{c'ij_{A}\overset{\wedge }{\Sigma }c'ij_{A}}},T_{\mathrm{ij}}^{B}=\frac{c'ij_{B}\bar{Y}}{\sqrt{c'ij_{B}\overset{\wedge }{\Sigma }c'ij_{B}}}$$

$\text{ where }\bar{Y}∼N(\mu ,\Sigma )\;\text{ and }\;\Sigma =\sigma^{2}D$

$$\begin{array}{c} D=[\begin{array}{c} 1/n_{11}\\ 1/n_{\mathrm{rs}} \end{array}]\\ T_{\mathrm{ij}}^{A}∼t_{n-1}(\delta_{A_{\mathrm{ij}}})\;\text{ with }\;\delta_{A_{\mathrm{ij}}}=\frac{\mu_{\mathrm{ij}}-\mu_{i0}}{\sigma \sqrt{(\frac{1}{n_{\mathrm{ij}}}+\frac{1}{n_{i0}})}},\\ T_{\mathrm{ij}}^{B}∼t_{n-1}(\delta_{B_{\mathrm{ij}}})\;\text{ with }\;\delta_{B_{\mathrm{ij}}}=\frac{\mu_{\mathrm{ij}}-\mu_{0j}}{\sigma \sqrt{(\frac{1}{n_{\mathrm{ij}}}+\frac{1}{n_{0j}})}},\\ \mathrm{Cov}(T_{\mathrm{ij}}^{A},T_{\mathrm{ij}}^{B})(\rho_{\mathrm{ij}})=\frac{1/n_{\mathrm{ij}}}{\sqrt{1/n_{\mathrm{ij}}+1/n_{i0}}\sqrt{1/n_{\mathrm{ij}}+1/n_{0j}}} \end{array}$$

where $t_{n-1}(\delta_{A_{\mathrm{ij}}})$ and $t_{n-1}(\delta_{B_{\mathrm{ij}}})$ are the non-central *t* distributions with non-centrality parameter $\delta_{A_{\mathrm{ij}}}$ and $\delta_{B_{\mathrm{ij}}}$, respectively, and with degrees of freedom $n-1=\sum_{\mathrm{ij}}n_{\mathrm{ij}}-1$.

The raw *p*-values (*p*_*ij*_) for testing *H*_0*ij*_ can be easily obtained but for testing multiple combinations; these raw *p*-values need to be adjusted. Multiplicity adjustment is challenging because the null distribution of the “Max” test statistic in equation (4) is unknown and it is difficult to compute. Essentially one needs to adjust the *p*_*ij*_ such that the FWER is controlled at significance level *α* for any of the possible null configurations. We can compute the *p*-values for all *H*_0*ij*_ and perform a Bonferroni correction for the (*r* × *s*) union tests shown in equation (3). However, the Bonferroni method is over conservative in almost all situations, so we consider alternative approaches to control the type 1 error rate. A more efficient approach is to control the maximum type 1 error based on the joint distribution of the test statistics. The maximum type 1 error can be calculated by searching for the “worst possible parameter configurations” within the null space for which the size of the test is maximized.^19^ We will see below that in our case the maximum type I error is not achieved by any finite parameter configurations but for hypothetical limiting cases where each *T*_*ij*_ becomes equal to either $T_{\mathrm{ij}}^{A}$ or $T_{\mathrm{ij}}^{B}$ because the other test statistics becomes infinitely large. This is further elaborated in the next paragraph. These limiting configurations are denoted by least favorable configurations (LFC) in the rest of the article. Since the LFC are impossible in reality, we suggest an alternative approach, where the mean parameters (μ) are estimated under the null constraint (3) and these null space restricted estimates are utilized via bootstrapping to obtain the critical value for the above multiple testing problem. This method gives more realistic estimates of the type 1 error rate. Note that the above multiple testing procedure is based on test statistics that satisfy the subset pivotality condition.^17^ The subset pivotality conditions asserts; if K⊂G, where *G* denotes the set of all active dose combinations and *K* is a subset of *G* where *H*_0*ij*_ is true for all (*i, j*) ∈ *K*, then the test statistics *T*_*ij*_, for some (*i, j*) ∈ *K* depend on the nuisance parameter $\left(\delta_{A_{\mathrm{ij}}},\delta_{B_{\mathrm{ij}}}\right)$ and sample sizes *n*_*ij*_, *n*_*i*0_ and *n*_0*j*_ but not on the remaining parameters $\{(\delta_{A_{\mathrm{ij}}},\delta_{B_{\mathrm{ij}}})\}(i,j)\notin K$. The distribution of $\max_{(i,j)\in K}T_{\mathrm{ij}}$ is therefore the same under the complete hypothesis $H_{0}^{G}=\cap_{(i,j)\in G}H_{0\mathrm{ij}}$ and the reduced hypothesis $H_{0}^{K}=\cap_{(i,j)\in K}H_{0\mathrm{ij}}$. Furthermore, each hypothesis is tested with a “Max” statistics and according to Westfall and Troendle^20^ both approaches, LFC and bootstrap approach, attain strong control of FWER. The following section elaborates how the multiplicity issue is dealt in the above testing problem.

#### 3.1.1 Least favorable null

The least favorable null configurations (LFC) identify the “worst case scenarios” that lead to the maximum type 1 error rate over the full parameter space. Note that the test statistics for evaluating *H*_0*ij*_, *T*_*ij*_, is stochastically bounded by both $T_{\mathrm{ij}}^{A}$ and $T_{\mathrm{ij}}^{B}$. Thus $P(T_{\mathrm{ij}}\geq t)\leq \min\{P(T_{\mathrm{ij}}^{A}\geq t),P(T_{\mathrm{ij}}^{B}\geq t)\}$ is maximum when equality holds, i.e. when *T*_*ij*_ attains one of the bounds. For evaluating the single hypothesis *H*_0*ij*_, the above situation arises when the combined mean *μ*_*ij*_ is equal to one of the monotherapies and infinitely larger than the other monotherapy. Hence, we conclude that the LFC for *H*_0*ij*_ occurs under such situations. This LFC can be best formulated as

$$\mathrm{LF}C_{\mathrm{ij}}={\mathrm{LFC}}_{\mathrm{ij}}^{A}\cup {\mathrm{LFC}}_{\mathrm{ij}}^{B}$$

where ${\mathrm{LFC}}_{\mathrm{ij}}^{A}=\{\mu_{\mathrm{ij}}=\mu_{i0}\;\text{ and }\;\mu_{\mathrm{ij}}>>\mu_{0j}\}$ and ${\mathrm{LFC}}_{\mathrm{ij}}^{B}=\{\mu_{\mathrm{ij}}>>\mu_{i0}\;\text{ and }\;\mu_{\mathrm{ij}}=\mu_{0j}\}$. Here *a* ≫ *b* indicates that *a* is infinitely larger than *b*. Following from here, the LFC for *H*_0_ in equation (3) occurs when the mean response *μ*_*ij*_ under each combination (*i, j*) is equal to one of the monotherapies and infinitely larger than the other. This can be written as

$$\mathrm{LFC}=\cap_{(i,j)\in K}({\mathrm{LFC}}_{\mathrm{ij}}^{A}\cup {\mathrm{LFC}}_{\mathrm{ij}}^{B})=\cup_{\tau \in {\left\{A,B\right\}}^{K}}\mathrm{LF}C_{\tau }=\cup_{\tau \in {\left\{A,B\right\}}^{K}}\cap_{(i,j)\in K}{\mathrm{LFC}}_{\mathrm{ij}}^{\tau_{\mathrm{ij}}}$$

where K={1,…,r}×{1,…,s} and *τ* ∈ {*A, B*}^*K*^ means that, *τ* is a map from *K* to {*A, B*}, i.e.

$$\tau :(i,j)\in K\to \tau_{\mathrm{ij}}\in \{A,B\}$$

We will call *τ* a configuration of *A*'s and *B*'s. Now, some *τ* ∈ {*A, B*}^*K*^ are infeasible in the sense that

$$\mathrm{LF}C_{\tau }:=\cap_{(i,j)\in K}{\mathrm{LFC}}_{\mathrm{ij}}^{\tau_{\mathrm{ij}}}=Ø$$

The set of all infeasible *τ* is given by

$$\begin{array}{c} \tau_{Ø}=\{\tau |\exists i\neq l,j\neq k,\text{suchthat}\tau_{\mathrm{ij}}=A,\\ \tau_{\mathrm{ik}}=B,\tau_{\mathrm{lj}}=B,\tau_{\mathrm{lk}}=A,\tau_{\mathrm{rem}}\in \{A,B\}K'\} \end{array}$$

where K'={K∖{(i,j),(i,k),(l,j),(l,k)}} and *τ*_*rem*_ is a map from K' to {A,B}K'. As one of the reviewers have pointed out, it might be interesting to explore the size of the infeasible LFC as compared to the set of feasible ones. We have written a R code to visualize the cardinality of $\tau_{Ø}$ for different choices of dose levels of Drug A (*r*) and dose levels of Drug B (*s*). It is added to the supplementary material. Table 1 shows the cardinality of $\tau_{Ø}$ for the different choices of dose levels of Drug A and Drug B. It is evident from the table that the rate of increase of infeasible LFC is very high as the number of dose levels increases. With the help of an illustrative example, we have shown later the set of infeasible LFC (2 out of 16) in a 3 × 3 drug combination trial, i.e. where *r* = 2 and *s* = 2. Furthermore, since we are interested in drug combination studies for Phase II clinical trials, the dose levels of the two drugs are unlikely to go beyond 4 or 5.

**Table 1.** Cardinality of infeasible LFC and the cardinality of all the LFC (in bracket) for different choices of dose levels of Drug A and Drug B, *r* and *s*, respectively.

	s			
r	1	2	3	4
1	0 (2)	0 (4)	0 (8)	0 (16)
2	0 (4)	2 (16)	18 (64)	110 (256)
3	0 (8)	18 (64)	282 (512)	3030 (4096)
4	0 (16)	110 (256)	3030 (4096)	58634 (65536)

Consider the critical value *C*_*α*_ for the above approach such that it satisfies

(5) $$\max_{\tau \in {\left\{A,B\right\}}^{K}\setminus \tau_{Ø}}(1-Pr_{\tau }(T_{11}\leq C_{\alpha },\ldots ,T_{r+1s+1}\leq C_{\alpha }))=\alpha$$

We reject the global null in equation (3) when the observed *T* > *C*_*α*_. Furthermore, all the component test statistics *T*_*ij*_ are tested against the critical value *C*_*α*_ and decisions are taken following a single-step testing procedure.^21^ The adjusted *p*-value for each component hypothesis *H*_0*ij*_ for the LFC approach can be obtained by computing the following probability; $\max_{\tau \in {\left\{A,B\right\}}^{K}\setminus \tau_{Ø}}\{P_{\tau }(\max_{i,j}T_{\mathrm{ij}}\geq t_{\mathrm{ij}})\}$, where *t*_*ij*_ is the observed value of the test statistics *T*_*ij*_ in equation (4). It is interesting to note in Table 1 that for a 5 × 5 combination trial, where *r* = 4 and *s* = 4 approximately 90% of the observed LFC are infeasible. One needs to compute the type 1 error only under 10% LFC and type 1 error computation is less time consuming under such a scenario as compared to computing the type 1 error under all possible LFC. The “Max” test together with the earlier mentioned subset pivotality property^22^ ensures that the FWER is controlled in the strong sense in the above testing approach. The following example shows an illustration of how one can obtain the maximum type 1 error using the LFC approach with four active drug combinations.

#### 3.1.2 Example

We consider a 3 × 3 drug combination study, i.e. a study with two drugs and two active doses per drug. Then $T=\max\{T_{11},T_{12},T_{21},T_{22}\}$, where $T_{\mathrm{ij}}=\min\{T_{\mathrm{ij}}^{A},T_{\mathrm{ij}}^{B}\}$ is the test statistics for comparing the *ij*^*th*^ drug combination with its monotherapies. The formal set of LFC is given in Table 2. In Table 2, *LF*_1_ denotes the set of dose combinations where the dose response means are equal to their first monotherapies and infinitely larger than their second monotherapies. *LF*_7_ and *LF*_10_ are marked grey because they are infeasible. The reason why *LF*_7_ is infeasible is that, ${\mathrm{LFC}}_{11}^{A}\Rightarrow \mu_{11}=\mu_{10},\mu_{11}>>\mu_{01}$

$$\begin{array}{c} {\mathrm{LFC}}_{12}^{B}\Rightarrow \mu_{12}>>\mu_{10},\mu_{12}=\mu_{02},\\ {\mathrm{LFC}}_{21}^{B}\Rightarrow \mu_{21}>>\mu_{20},\mu_{21}=\mu_{01},\\ {\mathrm{LFC}}_{22}^{A}\Rightarrow \mu_{22}=\mu_{20},\mu_{22}>>\mu_{02} \end{array}$$

**Table 2.** All least favorable null configurations for a 3 × 3 drug combination trial with four dose combinations.

Least Favorable Null	(1.1)	(1.2)	(2.1)	(2.2)
*LF*_1_	${\mathrm{LFC}}_{11}^{A}$	${\mathrm{LFC}}_{12}^{A}$	${\mathrm{LFC}}_{21}^{A}$	${\mathrm{LFC}}_{22}^{A}$
*LF*_2_	${\mathrm{LFC}}_{11}^{A}$	${\mathrm{LFC}}_{12}^{A}$	${\mathrm{LFC}}_{21}^{A}$	${\mathrm{LFC}}_{22}^{B}$
*LF*_3_	${\mathrm{LFC}}_{11}^{A}$	${\mathrm{LFC}}_{12}^{A}$	${\mathrm{LFC}}_{21}^{B}$	${\mathrm{LFC}}_{22}^{A}$
*LF*_4_	${\mathrm{LFC}}_{11}^{A}$	${\mathrm{LFC}}_{12}^{A}$	${\mathrm{LFC}}_{21}^{B}$	${\mathrm{LFC}}_{22}^{B}$
*LF*_5_	${\mathrm{LFC}}_{11}^{A}$	${\mathrm{LFC}}_{12}^{B}$	${\mathrm{LFC}}_{21}^{A}$	${\mathrm{LFC}}_{22}^{B}$
*LF*_6_	${\mathrm{LFC}}_{11}^{A}$	${\mathrm{LFC}}_{12}^{B}$	${\mathrm{LFC}}_{21}^{A}$	${\mathrm{LFC}}_{22}^{B}$
*LF*_7_	${\mathrm{LFC}}_{11}^{A}$	${\mathrm{LFC}}_{12}^{B}$	${\mathrm{LFC}}_{21}^{B}$	${\mathrm{LFC}}_{22}^{A}$
*LF*_8_	${\mathrm{LFC}}_{11}^{A}$	${\mathrm{LFC}}_{12}^{B}$	${\mathrm{LFC}}_{21}^{B}$	${\mathrm{LFC}}_{22}^{B}$
*LF*_9_	${\mathrm{LFC}}_{11}^{B}$	${\mathrm{LFC}}_{12}^{A}$	${\mathrm{LFC}}_{21}^{A}$	${\mathrm{LFC}}_{22}^{A}$
*LF*_10_	${\mathrm{LFC}}_{11}^{B}$	${\mathrm{LFC}}_{12}^{A}$	${\mathrm{LFC}}_{21}^{A}$	${\mathrm{LFC}}_{22}^{B}$
*LF*_11_	${\mathrm{LFC}}_{11}^{B}$	${\mathrm{LFC}}_{12}^{A}$	${\mathrm{LFC}}_{21}^{B}$	${\mathrm{LFC}}_{22}^{A}$
*LF*_12_	${\mathrm{LFC}}_{11}^{B}$	${\mathrm{LFC}}_{12}^{A}$	${\mathrm{LFC}}_{21}^{B}$	${\mathrm{LFC}}_{22}^{B}$
*LF*_13_	${\mathrm{LFC}}_{11}^{B}$	${\mathrm{LFC}}_{12}^{B}$	${\mathrm{LFC}}_{21}^{A}$	${\mathrm{LFC}}_{22}^{A}$
*LF*_14_	${\mathrm{LFC}}_{11}^{B}$	${\mathrm{LFC}}_{12}^{B}$	${\mathrm{LFC}}_{21}^{A}$	${\mathrm{LFC}}_{22}^{B}$
*LF*_15_	${\mathrm{LFC}}_{11}^{B}$	${\mathrm{LFC}}_{12}^{B}$	${\mathrm{LFC}}_{21}^{B}$	${\mathrm{LFC}}_{22}^{A}$
*LF*_16_	${\mathrm{LFC}}_{11}^{B}$	${\mathrm{LFC}}_{12}^{B}$	${\mathrm{LFC}}_{21}^{B}$	${\mathrm{LFC}}_{22}^{B}$

$\Rightarrow \mu_{10}=\mu_{11}>>\mu_{01}=\mu_{21}>>\mu_{20}=\mu_{22}>>\mu_{02}=\mu_{12}>>\mu_{10}$, which gives a contradiction. Similarly *LF*_10_ leads to another contradiction and thus it is infeasible as well.

The maximum type 1 error can be computed as

$$\begin{array}{c} \max\{1-\mathrm{Pr}(T_{11}^{A}\leq c,T_{12}^{A}\leq c,T_{21}^{A}\leq c,T_{22}^{A}\leq c),\\ 1-\mathrm{Pr}(T_{11}^{A}\leq c,T_{12}^{A}\leq c,T_{21}^{A}\leq c,T_{22}^{B}\leq c),\\ 1-\mathrm{Pr}(T_{11}^{A}\leq c,T_{12}^{A}\leq c,T_{21}^{B}\leq c,T_{22}^{A}\leq c),\\ ,\\ 1-\mathrm{Pr}(T_{11}^{B}\leq c,T_{12}^{B}\leq c,T_{21}^{B}\leq c,T_{22}^{B}\leq c)\} \end{array}$$

where each probability within the max is attained under each LFC in Table 2.

Obviously the FWER is bounded by the maximum over all the *LF*_*i*_ in Table 2, but note that we have omitted the infeasible *LF*_7_ and *LF*_10_. We will now show that the FWER under all mean constellations are controlled by the feasible LFC (LFC obtained after omiting *LF*_7_ and *LF*_10_). Clearly, the FWER under any mean constellation increases when replacing inequalities *μ*_*ij*_ ≤ *μ*_*i*0_ and *μ*_*ij*_ ≤ *μ*_0*j*_ by equalities. The resulting FWER is then dominated by the *LF*_*i*_ in Table 2 with similar equalities, i.e. the FWER under any mean constellation with *μ*_*ij*_ ≤ *μ*_*i*0_ is bounded by the corresponding FWER under the LFC where *μ*_*ij*_ = *μ*_*i*0_ (LFC where *τ*_*ij*_ = *A*), and the FWER under any mean constellation with *μ*_*ij*_ ≤ *μ*_0*j*_ is bounded by the corresponding FWER under the LFC where *μ*_*ij*_ = *μ*_0*j*_ (LFC where *τ*_*ij*_ = *B*). Assume now that *μ* corresponds to the set similar to *LF*_7_ where the dose combination means are equal to one monotherapy and finitely larger than the other monotherapy. We call this case

$$\begin{array}{c} F=\{\mu_{11}=\mu_{10},\mu_{11}\geq \mu_{01},\mu_{12}\geq \mu_{10},\mu_{12}=\mu_{02},\mu_{21}\geq \mu_{20},\mu_{21}=\mu_{01},\mu_{22}=\mu_{20},\mu_{22}\geq \mu_{02}\} \end{array}$$

Since we are omitting *LF*_7_ which is a bound for the FWER under the mean constellation *F*, we explore other bounds for this within the FWER under the feasible *LF*_*i*_'s. Note that this particular configuration, *F*, can hold true only when all the inequalities are substituted by equality, i.e. *μ*_*ij*_ = *μ*_*i*0_ = *μ*_0*j*_ for all (*i, j*). Under this configuration, the FWER is bounded by the FWER of any feasible *LF*_*i*_ because the probability of rejection goes up when one of the means is really high. Hence controlling the maximum type 1 error within the significance level *α* ensures that the FWER is controlled in the strong sense.

**Figure 1.**
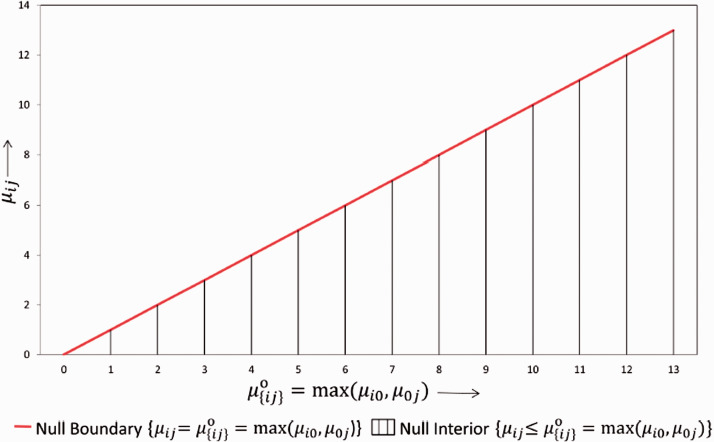
The null parameter space for any dose combination.

**Bootstrap test**: Under this approach, we aim to approximate the true null distribution of the test statistics using bootstrap methods. An illustration of the null parameter space is given in Figure 1. Here we estimate the parameters $\left\{\mu_{\mathrm{ij}}|i=0,\ldots r,j=0,\ldots s\right\}$ either under the null boundary constraint or under the space of all null configurations including the null interior constraint shown in Figure 1. With the bootstrap method, we generate samples under model (1) using estimates of the parameter *μ*_*ij*_ and *σ*^2^. These bootstrap samples are then utilized to obtain a sample of bootstrap test statistics with an empirical distribution, that is ideally a good approximation of the unknown underlying true null distribution of the test statistics T in equation (4). The parametric bootstrap approach can be outlined in more details by the following steps. Note that 2*a* and 2*b* below show the two options of projecting on the null space corresponding to the two different constraints illustrated in Figure 1.

1. For a given multiple dose combination study, compute the test statistics $T=\max_{i,j}\min\{T_{\mathrm{ij}}^{A},T_{\mathrm{ij}}^{B}\}$ where $T_{\mathrm{ij}}^{A}$ and $T_{\mathrm{ij}}^{B}$ are defined earlier in section 3.
2. For the given dataset estimate the mean under the constraints, i.e.
  a) $\mu_{\mathrm{ij}}=\max(\mu_{i0},\mu_{0j})\forall i,j$ such that, ${\overset{\wedge }{\mu }}_{a}={\mathrm{argmin}}_{\left\{\mu |\forall (i,j)\mu_{\mathrm{ij}}=\max(\mu_{i0},\mu_{0j})\right\}}\sum_{\left(i,j,k\right)}(Y_{\mathrm{ijk}}-\mu_{\mathrm{ij}})2$
  b) $\mu_{\mathrm{ij}}\leq \max(\mu_{i0},\mu_{0j})\forall i,j$ such that, ${\overset{\wedge }{\mu }}_{b}={\mathrm{argmin}}_{\left\{\mu |\forall (i,j)\mu_{\mathrm{ij}}\leq \max(\mu_{i0},\mu_{0j})\right\}}\sum_{\left(i,j,k\right)}(Y_{\mathrm{ijk}}-\mu_{\mathrm{ij}})2$ The standard deviation is estimated as $\frac{\left(Y-\overset{\wedge }{\mu })'(Y-\overset{\wedge }{\mu }\right)}{\sum_{i,j}n_{\mathrm{ij}}-(r+1)(s+1)}$, where $\overset{\wedge }{\mu }$ is the unrestricted maximum likelihood estimate (mle) of *μ*.
3. (*a*) Simulate 5000 normal distributed random variables with mean estimate ${\overset{\wedge }{\mu }}_{a}$ and (*b*) Simulate 5000 normal distributed random variables with mean estimates ${\overset{\wedge }{\mu }}_{b}$ from the earlier step. The standard deviation estimates remain the same in both the cases. For each simulated data compute the test statistics T in equation (4).
4. Find out the proportion of times the test statistics from the simulated data is greater than the observed test statistics both in case (*a*) and (*b*). This gives our *p*-value for the above bootstrap test under option (*a*) and (*b*), respectively.

Note that instead of calculating a *p*-value we could equivalently calculate the (1 – *α*) quantile of the bootstrap distribution for *T* and use this as the critical value for *T*. We will refer to this bootstrap critical value in the next section. We have used the unrestricted mle $\overset{\wedge }{\mu }$ to estimate $\overset{\wedge }{\sigma }$ in the above bootstrap approach instead of the restricted estimate in order to make sure that the bootstrapped test statistics satisfy the subset pivotality criteria mentioned earlier. This ensures in the above “Max” test, that the FWER is strongly controlled under the bootstrap approach as well.^20^ By this, we can not only claim that the drug-combination is beneficial overall but also provide the set of dose combinations showing beneficial effect in our study. We have observed in simulation studies that the option 2*a* provides better type 1 error control than option 2*b*. This is not surprising because the null hypotheses on the boundary are less favorable than those in the interior. In the numerical example and the simulation studies below, we present only the bootstrap approach under the option 2a.

### 3.2 Relationship between the different approaches

With the bootstrap approach, the critical value depends on the data via the constraint parameter estimates. Hence, the critical value is a random variable in the bootstrap approach whereas it is a fixed number for the LFC and Hung's approach.^7^

**Figure 2.**
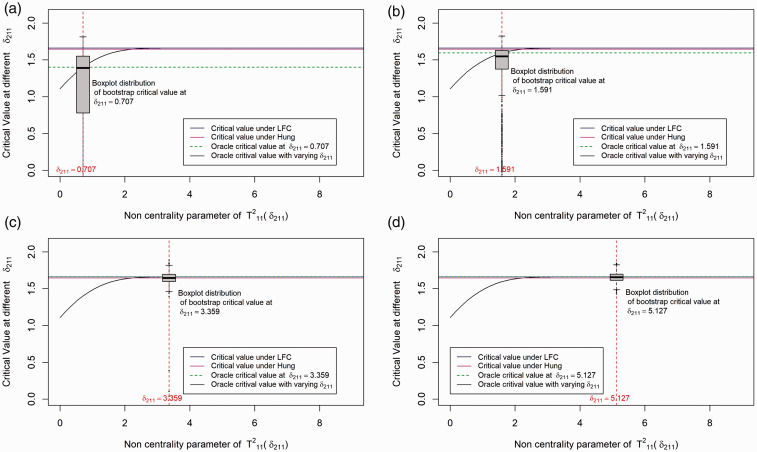
Distribution of critical value for the different methods under the set up: Drug A and Drug B both have one active dose group with sample size per dose group=25. The first effect size (*δ*_1_) is 0 and the second effect size (*δ*_2_) varies. Note that $T_{11}^{2}$ is addressed as $T_{11}^{B}$ in the article and the plots are shown in terms of the non-centrality parameter of test statistics $T_{11}^{2}$, i.e. $\delta_{211}=\delta_{2}/\sqrt{2/25}$, which is the second effect size scaled by the harmonic sum of sample size of the dose combination and the second monotherapy. (a) Boxplot distribution at *δ*_211_ = 0.707; (b) boxplot distribution at *δ*_211_ = 1.591; (c) boxplot distribution at *δ*_211_ = 3.359; (d) boxplot distribution at *δ*_211_ = 5.127.

In this section, we investigate different approaches by comparing their critical values under a particular dose combination setup. We illustrate the distribution of critical values for the different methods in Figure 2. For simplicity we simulate data from a balanced factorial design with *r* = 1, *s* = 1, and *n*_*ij*_ = 25 for all (*i, j*) combinations. Further we fix the first effect size at 0, i.e. set *δ*_1_ = *μ*_11_ – max(*μ*_10_, *μ*_01_) = 0 and the second effect size (*δ*_2_ = *μ*_12_ – max(*μ*_10_, *μ*_02_)) begins with 0 and increases along the X-axis. For the above setup, we plot the critical value of the test statistic (along the Y-axis) for the LFC approach, Hung's^7^ approach, Bootstrap approach and the oracle critical value, i.e. the critical value that can be derived if we know the unknown true null distribution of the test statistics. Since there is only one combination involved, computing the distribution of the test statistic under the global null *H*_0_ in equation (3) is not complicated. We observe that the critical value under the LFC is indeed a limiting case of the oracle critical values.

Our LFC approach is similar to the one in Hung's^7^ approach with the exception that it does not rely on an asymptotic normal approximation. The LFC approach identifies the same configurations as the extreme parameter configurations of the parameter *δ*_*ij*_ (where $\left|\delta_{\mathrm{ij}}|=|\mu_{i0}-\mu_{0j}\right|$) introduced by Hung.^7^ Under these extreme configurations using multiple testing theory, we show that the LFC leads to a multivariate *t* distribution for the test statistics *T* in equation (4) under the null *H*_0_ in equation (3). It is likely that the asymptotic approximation was introduced by Hung^7^ because numerical techniques for efficient evaluation of probabilities over rectangular region^23^ were not available then. Nevertheless, our illustration for a 2 × 2 factorial design in Figure 2 shows that the difference in the critical value between the two methods is marginal, with the LFC being more conservative than Hung's approach.

We have conducted 1000 simulations at some particular fixed values of *δ*_1_ and *δ*_2_. *δ*_1_ is fixed at 0 and 4 different values of *δ*_2_ are selected and the boxplots distribution of the critical values for the bootstrap method under the different values of *δ*_2_ is shown in Figure 2. As one can see from the plot, the critical value under the bootstrap method is approximately centered around the oracle critical value at each pre-selected *δ*_2_ (shown by the dotted green line). However, there might be cases where the critical value under the bootstrap method is smaller or larger compared to the oracle critical value. Asymptotically the type error should be controlled strongly with the subset pivotality criteria^20^ under the bootstrap approach but the critical value is sometimes underestimated as shown in Figure 2(c), thereafter explaining the inflation in type 1 errors observed in our simulation scenarios shown in Table 15.

**Figure 3.**
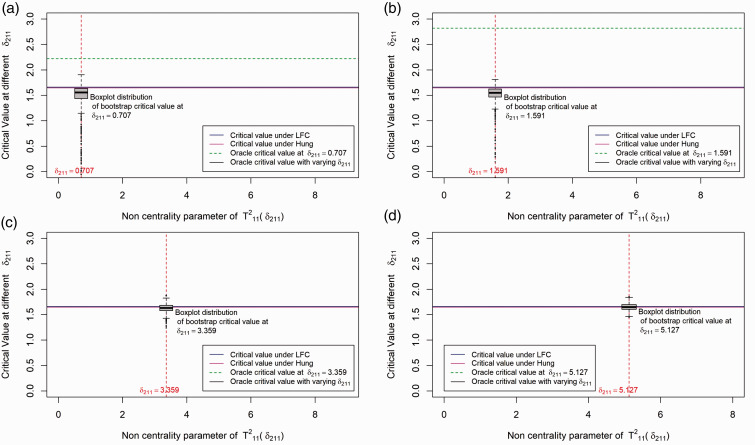
Distribution of critical value for the different methods under the set-up: Drug A and Drug B both have one active dose group with sample size per dose group = 25. The first effect size (*δ*_1_) is 0.5 and the second effect size (*δ*_2_) varies along X-axis. Note that $T_{11}^{2}$ is addressed as $T_{11}^{B}$ in the article and the plots are shown in terms of the non-centrality parameter of test statistics $T_{11}^{2}$, i.e. $\delta_{211}=\delta_{2}/\sqrt{2/25}$, which is the second effect size scaled by the harmonic sum of sample size of the dose combination and the second monotherapy. This plot is provided to analyze the empirical power performance of the different methods. (a) Boxplot distribution at *δ*_211_ = 0.707; (b) boxplot distribution at *δ*_211_ = 1.591; (c) boxplot distribution at *δ*_211_ = 3.359; (d) boxplot distribution at *δ*_211_ = 5.127.

A similar illustration of the above scenario with *δ*_1_≠0 is given in Figure 3 of Appendix 1. In summary, it is observed that the critical value under the bootstrap method is always centered around the least favorable null critical value and it is with a high probability below the least favorable critical value for smaller *δ*_2_. This indicates that the bootstrap method will give more power than the LFC approach.

From the above illustration, it is expected that the LFC approach gives more conservative results compared to the bootstrap approach and it also shows that Hung's^7^ approach behaves similar to the LFC approach.

## 4 Numerical example

We consider here an example from Hung^7^ to illustrate the methods discussed in the previous sections. A combination of a diuretic (drug B) and an ACE inhibitor (drug A) is tested for the efficacy in reduction of sitting diastolic pressure (SiDBP) with a pooled standard deviation of *σ* = 7.07. The response means and sample sizes are summarized in Table 3.

**Table 3.** Mean responses and the sample sizes (in bracket) of the drug combination study.

	Drug B		
Drug A	0	1	2
0	0 (75)	1.8 (74)	2.8 (48)
1	1.4 (75)	2.8 (75)	4.5 (50)
2	2.7 (74)	5.7 (74)	7.2 (48)
3	4.6 (48)	8.2 (49)	10.9 (48))

In total 750 patients are randomized to receive one of the 12 dose combinations. The primary objective of the study is to test whether there exist at least a combination (*i, j*) which is superior to the component drugs. Table 4 shows the unadjusted *p*-values as well as the adjusted *p*-values from the different methods for each dose combination.

**Table 4.** Unadjusted and adjusted p-values for each drug combination, when different methods are applied to the data example in Table 3.

Dose Comb	TStat	UnadjP	BonfP	BootP	LFCP
(1, 1)	0.863	0.194	1.000	0.650	0.709
(1, 2)	1.190	0.117	0.703	0.452	0.512
(3, 1)	2.507	0.006	0.037	0.024	0.036
(2, 1)	2.581	0.005	0.030	0.020	0.029
(2, 2)	3.049	0.001	0.007	0.004	0.007
(3, 2)	4.365	0.000	0.000	0.000	0.000

Dose Comb: Different dose combinations TStat : Test statistics T_ij_ testing for H_0ij_. UnadjP is the one-sided raw p-value testing H_0ij_ against H_1ij_. BonfP, BootP and LFCP are the one sided Bonferroni adjusted, Bootstrap adjusted and LFC adjusted p-values respectively.

We see from Table 4 that all the approaches proposed by us suggest that at 5% significance level the combinations (2, 1), (2, 2), (3, 1), and (3, 2) are superior to the monotherapies. For the Bonferroni adjusted method, the *p*-values are compared to the local significance level 0.8% to control the overall type 1 error at 5%. This numerical example was also investigated by other authors. Based on a global test, Hung^7^ concluded that there exists at least one combination that is superior to the monotherapies. He also applied an approximation of James^24^ to adjust the *p*-values and thereby identified the dose combination (2, 1), (2, 2) and (3, 2) as superior. But now that there is no need to approximate the distribution of the test statistics because Genz and Bretz^23^ proposed numerical techniques for an efficient evaluation of multivariate *t*-distribution probabilities over rectangular regions. Hellmich and Lehmacher^8^ implemented the AVE and MAX test proposed by Hung^7^ using the above proposed adjustment on the same dataset and concluded that the combination (2, 2) and (3, 2) are superior at 2.5% significance level (one-sided) with strong FWER control. Furthermore, using Holm's method, they showed that dose combinations (2, 1) and (3, 1) are also effective at significance level 2.5% (one-sided) to control the FWER at 5%. Soulakova^12^ cited the same example and concluded using Holm's approach that all the four combinations: (2, 1), (2, 2), (3, 1), and (3, 2) are superior and effective at significance level 5%. However, unlike Hellmich and Lehmacher,^8^ they conducted a test for both effectiveness and superiority and did not assume a priori that the dose combinations means are always greater than or equal to the monotherapy means.

## 5 Simulation studies

In this section, a simulation study is presented. The main objective of this simulation study is to compare the performance of the following approaches: (1) Hung's method, (2) parametric bootstrap method, (3) Bonferroni correction and (5) LFC approach with regards to their (*a*) ability to control the overall type 1 error at 5% significance level and (*b*) power to detect the superior dose combinations. We consider 11 scenarios overall, amongst which Scenario 1 and Scenario 2 are designed to investigate the strong control of type 1 error rate and Scenario 3 to Scenario 11 are designed to compare power performance of the different testing strategies. In Scenario 1 to Scenario 3 we are considering a balanced factorial design with *r* = 2, *s* = 1, and *n*_*ij*_ = *n* for all (*i, j*) combinations. In Scenario 4 to Scenario 11 we are considering an unbalanced factorial design with *r* = 3, *s* = 2, and differing *n*_*ij*_ for the (*i, j*) combinations.

### 5.1 Simulation scenarios

The simulation scenarios are divided into two parts. Section 5.1.1 refers to some new scenarios which are introduced in this article and Section 5.1.2 refers to the scenarios taken from Hung (2000).^7^

#### 5.1.1 New scenarios

We are considering three scenarios. Scenario 1 refers to an extreme situation where the parameters are drawn from the restricted parameter space *LFC*_feasible_. Note that in Table 5 where we are presenting Scenario 1, we assign *δ* = 2 and *a* = 9999, where 9999 represents an essentially large number close to the LFC where some dose combination means are infinitely larger than those of the monotherapies. Scenario 1 is to evaluate the ability of the different methods in controlling the type 1 error rate under extreme situations. For this we simulate the data randomly from one of the four cases shown in Table 5, where each *LF*_*i*_ represents a configuration that can occur in a 3 × 2 factorial design:

**Table 5.** Four least favorable null configurations in a 3 × 2 factorial design.

Least Favorable Config	Drug A	Drug B	
		0	1
*LF*_4_	0	*δ*	*δ* + *a*
	1	*δ*	*δ* + *a*
	2	*δ*	*δ* + *a*
*LF*_3_	0	*δ*	*δ* + *a*
	1	*δ*	*δ* + *a*
	2	*δ* + 2*a*	*δ* + 2*a*
*LF*_2_	0	*δ*	*δ* + *a*
	1	*δ* + 2*a*	*δ* + 2*a*
	2	*δ*	*δ* + *a*
*LF*_1_	0	*δ*	*δ*
	1	*δ* + *a*	*δ* + *a*
	2	*δ* + *a*	*δ* + *a*

However, as Scenario 1 is very extreme, Scenario 2 is considered to evaluate the performance of the different methods under a more realistic set up, where the value of *a* in Scenario 1 is replaced by 0.7. To evaluate the power performance across different sample sizes in a balanced design, data are simulated under Scenario 3 shown in Table 6.

We consider five different sample sizes for the above described scenarios: 10, 25, 50, 75, 100. We evaluate the empirical type 1 error and power based on 5000 simulation runs assuming normally distributed errors with standard deviation 1. For the parametric bootstrap method, 5000 bootstrap samples are used.

#### 5.1.2 Scenarios from Hung (2000)

Scenarios 4–11 are reproduced from Hung (2000).^7^ They are introduced to assess the power performances of the different methods under different effect sizes. Effect sizes here indicate the value of the contrast comparing the dose combination with the best monotherapy (*θ*_*ij*_ = *μ*_*ij*_ – max(*μ*_*i*0_, *μ*_0*j*_)). We want to further investigate the power performance of the different methods under a balanced and unbalanced design. Two possible effect sizes (E1 and E2) are shown in Tables 7 and 8. The average effect size (average of *θ*_*ij*_) is 0.3 for both designs but in E1 all effect sizes is equal and in E2 the combination of the lower dose A1 has a smaller effect compared to E1. For the above two designs, we consider four possible sample size allocations (S1, S2, S3, S4) given in Tables 9 to 12. S1 is a balanced design. S2 is introduced to increase the power of dose combination (A2, B1). Here (A2, B1) denotes the dose combination with dose level 2 of Drug A and dose level 1 of Drug B. S3 is designed such that more sample size is allocated to monotherapies corresponding to Drug A. This is introduced to ensure sufficient power of the drug combinations when compared with the first monotherapies. S4 is introduced to ensure more power for combinations with higher doses, particularly when the data is simulated from E2.

**Table 6.** Scenario 3: A balanced design scenario devised to evaluate the power of the different methods across different sample sizes.

	Drug B	
Drug A	0	1
0	2	2
1	2	2.5
2	2	2.5

**Table 7.** Dose-response means for the factorial design E1.

	Drug B		
Drug A	B0	B1	B2
A0	0	0.2	0.5
A1	0.1	0.5	0.8
A2	0.3	0.6	0.8
A3	0.6	0.9	0.9

**Table 8.** Dose-response means for the factorial design E2.

	Drug B		
Drug A	B0	B1	B2
A0	0	0.2	0.5
A1	0.1	0.25	0.65
A2	0.3	0.70	0.90
A3	0.6	1.0	1.0

**Table 9.** Sample size scenario (S1) for the drug combination designs (E1 and E2).

	Drug B		
Drug A	B0	B1	B2
A0	50	50	50
A1	50	50	50
A2	50	50	50
A3	50	50	50

### 5.2 Simulation results

Tables 14 and 15 shows how the type 1 error rate is controlled under the different methods for Scenario 1 and Scenario 2, respectively. Table 16 presents the empirical power of the different approaches under Scenario 3. Table 17 presents the empirical power of the different approaches under each effect size pattern and each sample size allocation for scenarios in Table 13.

**Table 10.** Sample size scenario (S2) for the drug combination designs (E1 and E2).

	Drug B		
Drug A	B0	B1	B2
A0	50	90	35
A1	35	35	35
A2	90	90	35
A3	35	35	35

**Table 11.** Sample size scenario (S3) for the drug combination designs (E1 and E2).

	Drug B		
Drug A	B0	B1	B2
A0	50	20	20
A1	70	50	50
A2	70	50	50
A3	70	50	50

**Table 12.** Sample size scenario (S4) for the drug combination designs (E1 and E2).

	Drug B		
Drug A	B0	B1	B2
A0	50	56	56
A1	30	30	30
A2	58	58	58
A3	58	58	58

**Table 13.** Scenario 4–Scenario 11.

Scenario	Dose Response Design	Sample Size
Scenario 4	E1	S1
Scenario 5	E1	S2
Scenario 6	E1	S3
Scenario 7	E1	S4
Scenario 8	E2	S1
Scenario 9	E2	S2
Scenario 10	E2	S3
Scenario 11	E2	S4

**Table 14.** Empirical type 1 error rate for the different methods: Scenario 1.

Sample Size	Bonf	Hung	Boot	LFC
10	0.045	0.057	0.047	0.045
25	0.050	0.055	0.053	0.051
50	0.047	0.049	0.048	0.047
75	0.044	0.045	0.046	0.044
100	0.048	0.050	0.049	0.049

**Table 15.** Empirical type 1 error rate for the different methods: Scenario 2.

Sample Size	Bonf	Hung	Boot	LFC
10	0.038	0.050	0.058	0.038
25	0.049	0.053	0.061	0.049
50	0.047	0.049	0.050	0.047
75	0.043	0.045	0.047	0.044
100	0.048	0.050	0.049	0.048

**Table 16.** Empirical power of the 5% level max test for the different methods under Scenario 3.

Sample Size	Bonf	Hung	Boot	LFC
10	0.1488	0.1718	0.2192	0.1530
25	0.4330	0.4432	0.5104	0.4380
50	0.7886	0.7936	0.8358	0.7904
75	0.9288	0.9308	0.9450	0.9304
100	0.9800	0.9790	0.9840	0.9806

**Table 17.** Empirical power of the 5% level max test for the different methods under the data scenarios in Table 13.

Scenario	Dose Response Design	Sample Size	Bonf	Hung	Boot	LFC
Scenario 4		S1	0.5622	0.5818	0.6596	0.5690
Scenario 5	E1	S2	0.5626	0.5750	0.6466	0.5670
Scenario 6		S3	0.4160	0.4510	0.5478	0.4222
Scenario 7		S4	0.5794	0.5974	0.6702	0.5846
Scenario 8		S1	0.7214	0.7350	0.7968	0.7286
Scenario 9	E2	S2	0.7538	0.7750	0.8254	0.7570
Scenario 10		S3	0.6102	0.6362	0.7322	0.6154
Scenario 11		S4	0.7930	0.8030	0.8572	0.7966

From Tables 14 and 15 we observe that the Bonferroni method and the LFC approach show a more conservative behavior compared to Hung's and the bootstrap approach. Note that though the Bonferroni method is criticized quite often in the literature, it performs almost as good as the LFC approach in our simulations.

The type 1 error is somewhat inflated for small sample sizes (e.g. *n*_*ij*_ = 10, 25 ∀*i, j*) with the bootstrap method. Tables 16 and 17 indicate that the parametric bootstrap approach shows uniformly better power performance than the other methods across all the sample sizes. This is because, under the alternative scenarios, the critical value of the bootstrap method is mostly below the critical value of the LFC approach. This has been elaborated in Section 4. The power performance of Hung's^7^ approach is similar to the power performance of the LFC approach. This is because they are essentially the same, one using an approximate and the other the exact distribution of the same test statistics. Note that we have used only 5000 iterations for the bootstrap approach in our simulation studies but as the number of iteration increases the inflation in type error is expected to reduce. Nevertheless, there will still be some inflation regardless of the number of bootstrap samplings and as the number of bootstrap iteration increases the method becomes more time consuming and the improvement is not significant, so we adhered to 5000 iterations. Summing up, the bootstrap approach is giving power improvement by 6–10% over the other approaches across all the scenarios.

We further observe from Table 17 that we can gain in power by choosing an unbalanced design with more sample size allocation in the combination doses compared to the component doses. In S3 the power performance dropped as more sample size is allocated to the monotherapies of Drug A compared to the dose combinations, whereas in S4, the power across all the methods are becoming better when more sample size is allocated to higher dose combinations compared to the monotherapies. The marginal improvement of Hung's method over the LFC approach is due to the approximate nature of the first.

## 6 Discussion

We observe from our simulation experiments that both the bootstrap method and the LFC approach, proposed in this article, meet the nominal level for attaining the global null hypothesis if per group sample sizes are not too small (e.g. 50 or more). They also strongly control the FWER at the desired significance level. Since the LFC approach is conservative across all sample sizes, the bootstrap approach is providing more power compared to the LFC. The LFC and Bonferroni approach performs surprisingly similar to each other. This is likely due to the fact that the least favorable null configuration will be the one where the test statistics are either independent or have low correlation. This follows from Slepian's inequality^25^ which says that the type 1 error from a “max” test, like the ones discussed in this article, increases with the decrease in the correlation between the component test statistics that combine to generate the max test statistic. The ideal maxima will occur when all the test statistics are uncorrelated, which will lead to the similar type 1 error as the type 1 error under the Bonferroni adjustment. However, in reality when there are multiple dose combinations involved, it is improbable to achieve this ideal maxima and hence the LFC type 1 error will always be greater than or equal to the Bonferroni type 1 error. This is also evident in our simulations. Note that we have only tested for superiority in our null hypothesis in equation (3). In order to test for effectiveness, we only need to add one additional test per dose combination, namely the test against placebo, such that we have overall the union of an intersection of three (instead of two) tests. Moreover, it is unlikely that we have non-efficacy but superiority with regard to both monotherapies. Hence, we have only considered superiority in our testing problem.

Resampling-based bootstrap approaches have already been suggested by other authors.^15,26^ Soulakova^15^ proposed a resampling based framework for a multiple dose factorial design where she identified all the effective and superior combinations without any considerations of the role of the nuisance parameters (difference in monotherapies) in the resampling distribution. Accordingly, she observed family-wise error rate inflations in multiple situations. Frommolt and Hellmich^26^ addressed this issue by a resampling-based bootstrap approach, where the nuisance parameters are estimated and accounted for in the resampling approximation of the test statistic's null distribution. But this bootstrap-based testing performed similar to the Hung's approach. The virtue of the parametric bootstrap approach suggested in this article is that, unlike the earlier approaches, it is performing better than the Hung's^7^ approach under the alternative hypothesis. As one of the reviewers pointed out, a potential downside of the parametric bootstrap approach is that it relies on the normal distribution assumption, which may not be always true. However, it is only a concern for small sample sizes; for large sample sizes, one can easily show using the central limit theorem that the test statistics is approximately *t* distributed regardless of the underlying data distribution.

The methods suggested here only provide a set of superior dose combinations but do not propose an optimal dose for future use in the drug developments process. We also cannot infer anything beyond the observed doses if the nature of dose response relationship is not known a priori. Hence, there is a strong interest in estimating the dose–response relationship for the drug combination. Hung^5,27^ suggested a response surface methodology approach, where after attaining global superiority one can utilize the biological information on the drug combination study and apply a statistical model to estimate the relationship between the drug dosages and mean response. This approach helps us to obtain an optimal dose and make inference around this optimal dose in a multiple dose drug combination trial. But often it happens that the true dose–response pattern is not known and then choosing an appropriate dose response model becomes difficult. Hellmich and Lehmacher^8^ proposed a closed testing procedure for estimating the minimum effective dose and highest effective dose levels in a dose–response bi-factorial design but they had to assume monotonicity properties to obtain the likelihood ratio tests and multiple contrast tests for their proposed hypotheses. To the best of our knowledge, there exists no approach where one can simultaneously control the FWER and infer on the dose–response relationship in a multiple dose drug combination study without the monotonicity assumption. Hence, it might be interesting to extend our bootstrap-based multiple testing approach to a modelling framework.

**Table 18.** A multiple dose drug combination factorial design.

	Drug B					
Drug A	0	1	2	.	.	s
0	*μ*_00_	*μ*_01_	*μ*_02_	.	.	*μ*_0*s*_
1	*μ*_10_	*μ*_11_	*μ*_12_	.	.	*μ*_1*s*_
.	.	.	.	.	.	.
r	*μ*_*r*0_	*μ*_*r*1_	*μ*_*r*2_	.	.	*μ*_*rs*_

**Table 19.** $\mu_{\tau }$: The mean constellation matrix following from W'.

	Drug B							
Drug A	1	2	.	*s*_2_	.	*s*_1_	.	*s*
1	*μ*_10_	*μ*_10_	.	.	.	*μ*_10_	$\mu_{0s_{1}+1}$	*μ*_0*s*_
2	*μ*_20_	*μ*_20_	.	*μ*_20_	$\mu_{0s_{2}+1}$	.	.	*μ*_0*s*_
⋮	.	.	.	.	.	.	.	.
r	.	.	.	.	.	.	.	.

## Supplemental Material

Supplemental material for Testing multiple dose combinations in clinical trials Click here for additional data file. Supplemental Material for Testing multiple dose combinations in clinical trials by Saswati Saha, Werner Brannath and Björn Bornkamp in Statistical Methods in Medical Research

## Appendix 1

Details on the least favourable configurations: We have a (*r* + 1) × (*s* + 1) factorial design as shown in Table 18. Following section 3, the set of all LFC is formally given by

$$\mathrm{LFC}=\cap_{(i,j)\in K}({\mathrm{LFC}}_{\mathrm{ij}}^{A}\cup {\mathrm{LFC}}_{\mathrm{ij}}^{B})=\cup_{\tau \in {\left\{A,B\right\}}^{K}}\cap_{(i,j)\in K}{\mathrm{LFC}}_{\mathrm{ij}}^{\tau_{\mathrm{ij}}}$$

where *K* = {1,…, *r*} × {1,…, *s*} and *τ* ∈ {*A, B*}^*K*^ is a map from *K* to {*A, B*} already explained section 3. Proposition All possible combinations in *τ* are feasible except the case where

$$\mathrm{LF}C_{\tau }=\cap_{(i,j)\in K}{\mathrm{LFC}}_{\mathrm{ij}}^{\tau_{\mathrm{ij}}}=Ø.$$

The set of all infeasible *τ* is given by

$$\begin{array}{c} \tau_{Ø}=\{\tau |\exists i\neq l,j\neq k,\text{suchthat}\tau_{\mathrm{ij}}=A,\\ \tau_{\mathrm{ik}}=B,\tau_{\mathrm{lj}}=B,\tau_{\mathrm{lk}}=A,\tau_{\mathrm{rem}}\in \{A,B\}K'\} \end{array}$$

where K'={K∖{(i,j),(i,k),(l,j),(l,k)}} and *τ*_*rem*_ is a map from K' to {A,B}K'. Proof Every configuration *τ* can be visualized as a matrix of *A*'s and *B*'s. It is easy to see

$${\mathrm{LFC}}_{\mathrm{ij}}^{A}\cap {\mathrm{LFC}}_{\mathrm{ik}}^{B}\cap {\mathrm{LFC}}_{\mathrm{lj}}^{B}\cap {\mathrm{LFC}}_{\mathrm{lk}}^{A}=Ø\text{ if }\;i\neq l,j\neq k$$

This is evident because the above condition will lead to the following submatrix for *τ* which is infeasible

$$\begin{array}{c} {}[\begin{array}{ccc} j & k\\ i & A & B\\ l & B & A \end{array}]\Rightarrow [\begin{array}{ccc} j & k\\ i & \mu_{i0} & \mu_{0k}\\ l & \mu_{0j} & \mu_{l0} \end{array}]\mathrm{and}[\begin{array}{c} \mu_{i0}>>\mu_{0j}\\ \mu_{0j}>>\mu_{l0}\\ \mu_{l0}>>\mu_{0k}\\ \mu_{0k}>>\mu_{i0} \end{array}] \end{array}$$

It remains to show that the above constellation is also a necessity condition for infeasibility.

Consider any feasible constellation *τ*. Let us denote the corresponding matrix by *W*. We show that *τ* cannot contain any other infeasibility criteria except the one in *τ*_*∅*_ using the following conjectures: Claim 1 Here we propose that it is always possible to permute the rows and columns of *W* to obtain the following matrix W'

**Table:**

	1	2	.	.	*s*_1_	.	.	*s*
1	A	A	.	.	A	B	B	B
.	A	A	.	B	B	B	B	B
.	.	.	.	.	.	.	.	.
.	.	.	.	.	.	.	.	.
r	.	.	.	.	.	.	.	. where W' have *s*_1_ *A*'s followed by *s* – *s*_1_ *B*'s in the first row, *s*_2_ *A*'s followed by *s* – *s*_2_ *B*'s in the second row, and so on. Here *s*_1_, *s*_2_,…, *s*_*r*_ can be any numbers between 0 and *s* which satisfies the inequality: *s*_1_ ≥ *s*_2_ ≥ … ≥ *s*_*r*_. Note that the above permutation is possible because permuting the rows or columns of *W* will not have any impact on the distribution of the test statistics under the LFC *τ*. Claim 2 Here we show that the matrix W' will lead to the following mean constellations $\mu_{\tau }$ in Table 19 and the parameters in this constellation can be chosen to meet in the limit, the criteria of an LFC, i.e. each *μ*_*ij*_ equals the mean of one of the corresponding monotherapies and becomes infinitely larger for the other one. Furthermore, we will show that the absence of an infeasible submatrix in $\mu_{\tau }$ is sufficient for showing the feasibility. Proof of Claim 1 We conduct the following operations on *W* to obtain W':

Operation 1: Consider the row with maximum number of *A*'s in *W* and permute the rows to make it the first row. Similarly the row with the second highest number of *A*'s is brought to the second row and so on. By this, we obtain a matrix, where the number of *A*'s in a row is non-increasing from the first to the last row.

Operation 2: Followed by Operation 1, permute the columns of matrix *W* such that the first row will have all *A*'s in the beginning followed by all B's. This will lead to the following matrix

$$W\prime \prime =\begin{array}{c} {}[\begin{array}{cc} A1_{1\times s_{1}} & B1_{1\times s-s_{1}}\\ Y_{r-1\times s_{1}} & Z_{r-1\times s-s_{1}} \end{array}] \end{array}$$

where $A1_{1\times s_{1}}$ is a row vector of all *A*'s and $B1_{1\times s-s_{1}}$ is a row vector of all *B*'s. Due to construction, $Z_{r-1\times s-s_{1}}$ has *B*'s in all entries by the following argument: If say the *k*^*th*^ row of $Z_{r-1\times s-s_{1}}$ would have at least one *A*, then *W* is feasible (following the criteria in *τ*_*∅*_) only when the corresponding *k*^*th*^ row of $Y_{r-1\times s_{1}}$ have all entries *A*. However, this will lead to the contradiction that (*k* + 1)^*th*^ row of the above matrix W″ has more *A*'s than the first row. Hence $Z_{r-1\times s-s_{1}}$ has *B*'s in all entries. Inductively applying Operation 2 on $Y_{r-1\times s_{1}}$ and thereafter on submatrices of $Y_{r-1\times s_{1}}$ will lead to the matrix W'.

Proof of Claim 2 It is evident that the permuted matrix W' in Claim 1 will lead to the mean constellation matrix $\mu_{\tau }$ in Table 19. It remains to show that the absence of an infeasible 2 × 3 submatrix in $\mu_{\tau }$ is sufficient for showing the feasibility.

We can conclude the following from the LFC criteria: *A* denotes that the first monotherapy mean is infitely larger than the second monotherapy mean and *B*, the vice versa. Hence from W' it follows, $\mu_{0j}<<\mu_{10}<<\mu_{0i}\forall j=1,\ldots ,s_{1},i=s_{1}+1,\ldots ,s$ and $\mu_{0j}<<\mu_{20}<<\mu_{0i}\forall j=1,\ldots ,s_{2},i=s_{2}+1,\ldots ,s$. Similar restrictions hold for the other rows in the above matrix. When *s*_*l*_≠0 for any *l*, this will lead to the following ordering between the means

(6) $$\begin{array}{c} \mu_{0i_{1}}>>\mu_{10}>>\mu_{0i_{2}}>>\mu_{20}\ldots >>\mu_{r-10}>>\mu_{0i_{r}}>>\mu_{r0}>>\mu_{0i_{r+1}} \end{array}$$

where $i_{1}\in \{s_{1}+1,\ldots ,s_{0}\},i_{2}\in \{s_{2}+1,\ldots ,s_{1}\},i_{3}\in \{s_{3}+1,\ldots ,s_{2}\}$ and continuing similarly $i_{r}\in \{s_{r}+1,\ldots ,s_{r-1}\}$ and $i_{r+1}\in \{s_{r+1}+1,\ldots ,s_{r}\}$ assuming *s*_0_ = *s* and *s*_*r* + 1_ = 0. Suppose *k* is the first row index such that *s*_*k*_ = 0, then *s*_*l*_ = 0,∀*l* ≥ *k* since *s*_*l*_ is a sequence of non-increasing numbers, i.e. *k*th row to *r*th row in the above matrix $\mu_{\tau }$ will have *B*'s in all entries. The above mean ordering then becomes

(7) $$\begin{array}{c} \mu_{0i_{1}}>>\mu_{10}>>\mu_{0i_{2}}>>\mu_{20}\ldots >>\mu_{0i_{k-1}}>>\mu_{k-10}>>\mu_{0i_{k}}\\ \text{ and }\;\mu_{k'0}<<\mu_{0j}\forall k'\in \{k,\ldots ,r\},j\in \{1,\ldots ,s\}\\ \Rightarrow \mu_{0i_{1}}>>\mu_{10}>>\mu_{0i_{2}}>>\mu_{20}\ldots >>\mu_{0i_{k-1}}>>\\ \mu_{k-10}>>\mu_{0i_{k}}>>\mu_{k'0},\forall k'\in \{k,\ldots ,r\} \end{array}$$

where *i*_1_,…,*i*_*k*_ are same as defined earlier. It can be easily seen that the above mean orderings can be realized by assuming that for all *l* ∈ {1,…, *r* + 1} the distinct monotherapy means $\left\{\mu_{0i_{l}},i_{l}\in \{s_{l}+1\ldots ,s_{l-1}\}\right\}$ are equal amongst themselves. Similar conclusions can be drawn in equation (8) by assuming $\left\{\mu_{0i_{l}},i_{l}\in \{s_{l}+1\ldots ,s_{l-1}\}\right\}$ are equal amongst themselves for any l∈{1,…,k} and $\left\{\mu_{k'0},k'\in k,\ldots ,r\right\}$ are equal amongst themselves. Hence, we have shown that the mean orderings in $\mu_{\tau }$ will not lead to any contradictions, thereby proving that the infeasibility criteria mentioned in *τ*_*∅*_ is the sufficient condition for infeasibility in the LFC approach.

## References

[1] Regulatory challenges for new formulations of controlled substances in today's environment. Drug Alcohol Dependence 2006; 83: S63–S67. https://www.sciencedirect.com/science/article/pii/S0376871606000548#bbib3. 16567060 10.1016/j.drugalcdep.2006.02.001

[2] Guidance for industry: codevelopment of two or more new investigational drugs for use in combination, https://www.fda.gov/downloads/drugs/guidances/ucm236669.pdf (2013).

[3] Laska EM Meisner MJ Testing whether an identified treatment is best. Biometrics 1989; 40: 1139–1151. . 2611321

[4] Snapinn SM Evaluating the efficacy of a combination therapy. Stat Med 1987; 6: 657–665. 2961039 10.1002/sim.4780060603

[5] Hung HJ Ng TH Chi GY, et al. Response surface and factorial designs for combination antihypertensive drugs. Drug Inform J 1990; 24: 371–378.

[6] Hung HJ Chi G Lipicky R Testing for the existence of a desirable dose combination. Biometrics 1993; 49: 85–94. . 8513112

[7] Hung HJ Evaluation of a combination drug with multiple doses in unbalanced factorial design clinical trials. Stat Med 2000; 19: 2079–2087. 10931512 10.1002/1097-0258(20000830)19:16<2079::aid-sim535>3.0.co;2-i

[8] Hellmich M Lehmacher W Closure procedures for monotone bi-factorial dose–response designs. Biometrics 2005; 61: 269–276. 15737103 10.1111/j.0006-341X.2005.030709.x

[9] Buchheister B Lehmacher W Multiple testing procedures for identifying desirable dose combinations in bifactorial designs. GMS Med Inform Biometry Epidemiol 2006; 2: 1–11.

[10] Soulakova JN Sampson AR On identifying minimum efficacious doses in combination drug trials. Stat Biopharmaceut Res 2009; 1: 39–47. 10.1198/sbr.2009.0004 PMC2724724 19675682

[11] Soulakova JN Comparison of several testing strategies for combination drug efficacy trials based on the closure principle. Stat Med 2009; 28: 260–273. 18991315 10.1002/sim.3462

[12] Soulakova JN On identifying effective and superior drug combinations via Holm's procedure based on the min tests. J Biopharmaceut Stat 2009; 19: 280–291. 10.1080/10543400802622469 19212880

[13] Soulakova JN General multistage gatekeeping procedures for identifying beneficial drug combinations in factorial trials with isotonic gains. Stat Biopharmaceut Res 2010; 2: 33–41.

[14] Holm S A simple sequentially rejective multiple test procedure. Scand J Stat 1979; 6: 65–70. .

[15] Soulakova JN Resampling-based and other multiple testing strategies with application to combination drug trials with factorial designs. Stat Meth Med Res 2011; 20: 505–521. 10.1177/0962280209105655 20667933

[16] Hochberg Y A sharper bonferroni procedure for multiple tests of significance. Biometrika 1988; 75: 800–802.

[17] Westfall PH, Young SS, et al. *Resampling-based multiple testing: examples and methods for p-value adjustment*, volume 279. Hoboken, NJ: John Wiley & Sons, 1993.

[18] Marcus R Eric P Gabriel KR On closed testing procedures with special reference to ordered analysis of variance. Biometrika 1976; 63: 655–660.

[19] Blanchard G, Dickhaus T, Roquain E, et al. On least favorable configurations for step-up-down tests. *arXiv preprint arXiv:11085262* 2011.

[20] Westfall PH Troendle JF Multiple testing with minimal assumptions. Biometric J: J Math Meth Biosci 2008; 50: 745–755. 10.1002/bimj.200710456 PMC3117234 18932134

[21] Tamhane AC Hochberg Y Dunnett CW Multiple test procedures for dose finding. Biometrics 1996; 52: 21–37. . 8934584

[22] Westfall PH Multiple testing of general contrasts using logical constraints and correlations. J Am Stat Assoc 1997; 92: 299–306.

[23] Genz A Bretz F Numerical computation of multivariate t-probabilities with application to power calculation of multiple contrasts. J Stat Computat Simulat 1999; 63: 103–117.

[24] James S Approximate multinormal probabilities applied to correlated multiple endpoints in clinical trials. Stat Med 1991; 10: 1123–1135. 1876800 10.1002/sim.4780100712

[25] Tong YL. *Probability inequalities in multivariate distributions*. Cambridge, MA: Academic Press, 2014.

[26] Frommolt P Hellmich M Resampling in multiple-dose factorial designs. Biometric J 2009; 51: 915–931. 10.1002/bimj.200900129 20029896

[27] Hung HJ Chi GY Lipicky RJ On some statistical methods for analysis of combination drug studies. Commun Stat Theory Meth 1994; 23: 361–376.

